# A phase I study of intravenous liposomal daunorubicin (DaunoXome) in paediatric patients with relapsed or resistant solid tumours

**DOI:** 10.1038/sj.bjc.6603288

**Published:** 2006-08-01

**Authors:** S Lowis, I Lewis, A Elsworth, C Weston, F Doz, G Vassal, R Bellott, J Robert, F Pein, S Ablett, R Pinkerton, D Frappaz

**Affiliations:** 1 Department of Oncology, Royal Hospital for Children, Maudlin Street, Bristol BS2 8BJ, UK; 2 St James's University Hospital, Leeds, UK; 3 United Kingdom Children's Cancer Study Group, University of Leicester, UK; 4 Institut Curie, Paris, France; 5 Institut Gustave Roussy, Villejuif, France; 6 Institut Bergonié, Bordeaux, France; 7 Royal Marsden Hospital, UK; 8 Centre Leon Berard, Lyon, France

**Keywords:** phase I, DaunoXome, cardiotoxicity, anthracycline, children, childhood cancer

## Abstract

Anthracyclines are widely used in paediatric oncology, but their use is limited by the risk of cumulative cardiac toxicity. Encapsulating anthracyclines in liposomes may reduce cardiac toxicity and possibly increase drug availability to tumours. A phase I study in paediatric patients was designed to establish the dose limiting toxicity (DLT) and maximum tolerated dose (MTD) after a single course of liposomal daunorubicin, ‘DaunoXome’, as a 1 h infusion on day 1 of a 21 day cycle. Patients were stratified into two groups according to prior treatment: Group A (conventional) and group B (heavily pretreated patients). Dose limiting toxicity was expected to be haematological, and a two-step escalation was planned, with and without G-CSF support. Pharmacokinetic studies were carried out in parallel. In all, 48 patients aged from 1 to 18 years were treated. Dose limiting toxicity was neutropenia for both groups. Maximum tolerated dose was defined as 155 mg m^−2^ for Group A and 100 mg m^−2^ for Group B. The second phase with G-CSF was interrupted because of evidence of cumulative cardiac toxicity. Cardiac toxicity was reported in a total of 15 patients in this study. DaunoXome shares the early cardiotoxicity of conventional anthracyclines in paediatric oncology. This study has successfully defined a haematological MTD for DaunoXome, but the significance of this is limited given the concerns of delayed cardiac toxicity. The importance of longer-term follow-up in patients enrolled into phase I studies has been underestimated previously, and may lead to an under-recognition of important adverse events.

Anthracycline antibiotics have a wide spectrum of activity, and are used in many adult and paediatric protocols for the treatment of haematological and solid malignancies. The risk of cardiomyopathy is dose-limiting, and is related to cumulative dose, young age, mediastinal radiotherapy and pre-existing cardiac impairment (Von Hoff *et al*, 1977, 1979; Steinherz *et al*, 1991; Pein *et al*, 2004). It may appear early or late, and major concerns have been raised by long-term follow-up studies of children treated early in their life (Poutanen *et al*, 2003; Pein *et al*, 2004). Strategies to reduce this risk have included limitation of the total administered dose, administration in fractionated rather than single doses, as infusions, the use of protective agents and liposomal encapsulation of the compound (Weiss and Manthel, 1977).

The effect of administration schedule of anthracyclines – as a bolus or as a prolonged infusion – has been studied in preclinical models and clinically. The pharmacokinetics of doxorubicin do appear to be affected by infusion duration (Cusack *et al*, 1993), with a reduction of cardiac exposure after a prolonged exposure (Cusack *et al*, 2003), and a beneficial effect of prolongation identified in some cases (Casper *et al*, 1991; Gupta *et al*, 2003) but not all (Speyer *et al*, 1985; Hortobagyi *et al*, 1989; Zalupski *et al*, 1991; Levitt *et al*, 2004). Similarly, the evidence for the use of cardioprotectants is mixed, and improved cardiotoxicity may be achieved at the expense of a reduced response rate (reviewed by van Dalen *et al* (2005)).

Liposomal encapsulation may lead to changes in the pharmacokinetics of the anthracycline, and this may increase delivery to the tumour and reduce that to healthy tissues. The anthracycline within DaunoXome (®, daunorubicin) is held within a vesicle comprising disteroyl phosphatidyl choline (DSPC) and cholesterol. Vesicles of this diameter (40–60 nm) are not rapidly cleared from the plasma by the reticulo-endothelial system, and release of daunorubicin continues in a sustained manner. Preclinical studies indicate increased tissue concentrations of daunorubicin in tumour, brain, liver, spleen and intestine following DaunoXome compared with free daunorubicin administration, but a reduced tissue concentration in cardiac tissue (AUC ratio 0.4) (Forssen and Ross, 1994). In addition, studies using radio-labelled vesicles suggest selective uptake into tumour (Forssen *et al*, 1992). An improved therapeutic index is therefore expected. In a murine lymphosarcoma model, significantly longer survival was seen in mice treated with DaunoXome than with free daunorubicin (Forssen and Ross, 1994).

Previous adult studies have reported acceptable toxicity with doses in excess of 100 mg m^−2^ (Eckardt *et al*, 1994; Guaglianone *et al*, 1994; Richardson *et al*, 1997; McBride *et al*, 2001). Most clinical studies with DaunoXome have been in adult patients, and cardiac toxicity has not been a prominent feature even with cumulative doses of over 1000 mg m^−2^, although follow-up of patients was short (Money-Kyrle *et al*, 1993; Presant *et al*, 1993; Guaglianone *et al*, 1994; Gill *et al*, 1995).

A French phase I study of DaunoXome in children with acute lymphoblastic leukaemia, delivered DaunoXome every 7 days for 4 weeks at a starting dose of 40 mg m^−2^ and did not reach an maximum tolerated dose (MTD). The maximum cumulative dose of anthracyclines was 720 mg m^−2^: only one patient, treated at the first level of 40 mg m^−2^, showed a decrease in fractional shortening of more than 10%. Owing to relapse or death, cardiac data are available in only 16 patients and all have normal function, although the assessment of cardiac function was made early post-treatment (A Baruchel, Personal communication).

A phase I study with a similar schedule in children with recurrent or progressive brain tumours reported encouraging activity (Lippens, 1999). Patients received DaunoXome as a 1 h infusion at a dose of 60 mg m^−2^, once every 4 weeks. Two patients had received prior anthracycline, but neither developed cardiac toxicity. Cardiac toxicity was seen in three of 14 children. One patient developed cardiac toxicity with a fall in FS to 20%. This patient had received prior radiotherapy to the spine (35 Gy), which had included some cardiac tissue, but toxicity was not identified until after the eighth course of DaunoXome. Two other patients developed transient reductions in FS to <28% after four and seven cycles, but in both, DaunoXome administration was continued and FS said to recover to normal values. An average transient reduction in the thickness of the posterior wall and septum was reported, but this too recovered within 6 months of cessation.

Overall, there seemed to be reasonable evidence to suggest that DaunoXome might offer good efficacy with acceptable toxicity. There seemed to be evidence of reduced cardiac toxicity to allow dosing beyond the conventional limit of 550 mg m^−2^, and we decided therefore to undertake a phase I study of DaunoXome in children with relapsed or resistant malignant solid tumours.

## STUDY AIMS

The primary aims of the study were to determine:

1. the dose limiting toxicity (DLT) and MTD of DaunoXome when given as a single infusion over 1 h to children with relapsed or resistant solid tumours in two cohorts, one having received conventional therapy and the other prior craniospinal radiotherapy or high-dose therapy;
2. a second DLT and MTD in each cohort of patients with G-CSF support using the MTD from the previous study;
3. the pharmacokinetics of liposomal daunorubicin in children.

The secondary aim was to document tumour response in these patients.

## STUDY DESIGN

### Patient eligibility

The study was open from December 1998 to June 2001 as a joint UK/French study. All centres participating were required to fulfil UKCCSG/SFOP criteria for phase I studies. Patients with recurrent or resistant malignant disease, aged between 1 and 18 years, with a predicted life expectancy of more than 8 weeks, and a performance status score (Karnofsky >30 or Lansky >3) were eligible for entry.

ECG, two-dimensional echocardiogram with documentation of Fractional Shortening and ejection fraction and a chest radiograph were recorded at entry. ECG and two-dimensional echocardiogram were repeated prior to each subsequent course of chemotherapy. Normal cardiac function, with a fractional shortening ⩾29% was required at entry into the study. A reduction of FS to <29% or a reduction of EF by more than 20% were used to define toxicity according to CTC criteria.

Laboratory investigations (creatinine, urea and electrolytes, calcium, magnesium, phosphate, alkaline phosphatase, alanine transaminase, albumin, bilirubin, full blood count with differential WBC and coagulation screen) were performed within 7 days of DaunoXome administration to ensure adequate haematological, renal and hepatic function, and were repeated prior to each subsequent course of chemotherapy.

Written informed consent was given by all parents or guardians, and by patients themselves where appropriate. In the UK, this study was approved by the Multi-Centre Research Ethics Committee for Scotland and by Local Research Ethics Committees of each participating centre. In France, approval was received from the CCPPRB (Comité Consultatif de Protection des Personnes dans la Recherche Biomedicale) with Centre Léon Bérard in Lyon acting as promoter. Study monitoring and Source Data Verification were performed according to ICH GCP guidelines.

### Study administration and drug supply

In all, 17 centres (12 UK, five French) were eligible to enter patients into the study. Coordination of the study was through the UKCCSG Data Centre. Once three patients were recruited at a dose level, recruitment was frozen to allow all those patients to complete their first cycle (30 days to allow for toxicities) and be monitored. Monitoring was organised by the UKCCSG Data Centre. DaunoXome was kindly supplied by Gilead Sciences (formerly NeXstar).

DaunoXome, was administered as a single, 1 h infusion on day 1. Where there was recovery from toxicity and no disease progression, treatment cycles were repeated every 21 days. In cases of DLT for the first cycle, patients were treated at the previous dose level for subsequent cycles. Patients were allowed up to a maximum of 12 courses of treatment.

### Recruitment/dose escalation

Two cohorts of patients were recruited, given an anticipated reduction in bone marrow reserve for patients who had undergone intensive chemotherapy

- Arm A – patients who relapsed after conventional therapy.
- Arm B – patients previously treated with craniospinal radiotherapy and/or high dose therapy.

High-dose therapy was defined as any procedure that required bone marrow or stem cell rescue after chemo- or radiotherapy. Patients were not stratified according to prior anthracycline exposure.

Dose limiting toxicity was defined from the safety profile of cycle one. No within-patient dose escalation was allowed, according to the method of Korn *et al* (1994). Three patients were recruited in each arm at each dose level until DLT was reached. If a single DLT was seen in one cohort, a maximum of three additional patients were recruited at that level. If DLT was not observed in these additional patients, then the next dose level was entered. The MTD was defined as that dose level immediately below the dose level at which a minimum of two patients in a cohort of three to six patients experienced DLT. A total of six patients were treated at the MTD to assess toxicity fully.

### Evaluation during treatment

Full blood counts and biochemistry tests including liver function were performed twice weekly (for courses 1 and 2, weekly for subsequent courses). Physical examination and vital signs were assessed weekly. Cardiac toxicity was assessed on day 21 of each cycle by Echo and ECG. Assessment of tumour burden was made at baseline, after two cycles, and after every other cycle. All imaging performed on any patient showing response was reviewed by two radiologists. The following definitions were used for response:

- Complete response (CR)=no radiological evidence of residual tumour on MRI scan,
- Partial response (PR)=>50% reduction of the product of two maximum perpendicular diameters of the tumour,
- Stable disease (s.d.)=<25% reduction of the product of two maximum perpendicular diameters of the tumour without radiological evidence of tumour progression or dissemination,
- Progressive disease (PD)=any radiological or clinical evidence of tumour progression, or any new tumour site.

#### Toxicity

Toxicity was graded according to the NCI Common Toxicity Criteria (CTC) 1998 (version 1) or, where this was not possible, according to severity (i.e. mild, moderate, severe or life threatening).

A DLT was defined during the 21 days after the first dose of DaunoXome as follows:

1. *Myelosuppression*: CTC grade IV neutropenia, thrombocytopenia or leucopenia *lasting at least 7 days*.
2. *Cardiac*: >20% fall in the ejection fraction at day 21 compared with initial values,
3. Clinically relevant nonhaematological CTC grade III or IV.

As a result of discussions regarding cardiac toxicity seen in some early patients, the definition of dose limiting cardiac toxicity was revised during the study. A single recorded fall of 20% in the ejection fraction at day 21 was defined initially as a DLT. Wide intrapatient variability was recorded in some patients, and concern was raised that an inappropriately low MTD may be identified if such changes were not representative of cardiac function. Indeed, two patients with a fall in ejection fraction at day 21 showed complete recovery of cardiac function at day 28. In consultation with cardiologists in France and the UK, a decision to require a sustained reduction in cardiac function (EF) of 20% was made, and therefore a repeat echocardiogram was performed at day 28 in any patient with a possible cardiac DLT. All cardiac toxicities were reported as Serious Adverse Events to the UKCCSG Data Centre.

Dose limiting toxicity and MTD were determined using DaunoXome alone, and subsequently with G-CSF support (Filgrastim 5 mcg kg^−1^ day^−1^). Patients with evidence of clinical progression discontinued after one course. Full evaluation of tumour response was undertaken after the second course. Patients with response or stable disease were eligible to continue DaunoXome for further disease assessment at the same dose, provided no DLT occurred. If a DLT occurred, patients were treated at one dose level below for subsequent cycles.

## RESULTS

### Patient characteristics

A total of 48 patients were enrolled into the study: an additional two patients were registered, but did not receive any treatment. Five patients were not evaluable for haematological toxicity at the end of cycle 1, but were eligible for cardiac toxicity. Three of the 48 patients were not evaluable for cardiac toxicity, because of failure to monitor after the first cycle of chemotherapy (patient unwell or refusal to allow monitoring).

Patient characteristics are summarized in Table 1. Arm A recruited more patients with brain tumours and a slight predominance of male patients (ratio of 1.4:1).

### Drug administration

Arm A patients were treated at five dose levels (dose (number)): 80 (2 – one patient eligible from arm B), 100 (3), 125 (8), 155 (7), 190 (7) mg m^−2^ per cycle (total 27 patients, 5 not fully evaluable). Arm B patients were treated at three dose levels, 80 (3), 100 (7), 125 (3) mg m^−2^ per cycle (total 13 patients, two not fully evaluable). Arm B patients with G-CSF were treated at 125 (3), 155 (3) and 190 (2) mg m^−2^ per cycle (total eight patients, one not fully evaluable). A total of 98 courses of DaunoXome were given in the whole study, a median of two courses per patient (range 1–8).

### Adverse events

An adverse event was defined as any CTC grade III or IV toxicity that occurred at any time while the patient was on study. Cardiac toxicity of any grade was reported. Serious adverse events were defined as:

- Death occurring within 30 days after the last study drug administration.
- Any event which was life threatening, incapacitating or permanently disabling, or required prolonged hospitaliation.
- Clinical or laboratory events which require withdrawal of the drug.

### Nonhaematological toxicities

Adverse events were reported for all children. In all, 38 reported adverse events as possibly, probably or definitely related to the study drug after 1 cycle.

A total of 260 nonhaematological adverse events (excluding cardiac toxicity) were reported during cycle 1. In all, 125 of these were classed as possibly, probably or definitely drug related (Table 2).

A total of 20 nonhaematological SAEs classed as possibly, probably or definitely related were reported in 17 patients during cycle 1. Many of these were related to coincident haematological toxicity.

Two patients developed allergic reactions to DaunoXome at the time of drug administration during cycle 1. Both patients developed a high temperature, one developed a generalised rash, and one developed chest tightness and flushing. In both cases, the drug was later restarted without further adverse event. One other patient developed a high temperature and a generalised maculo-papular exanthema shortly after the start of treatment during cycle 2. These symptoms disappeared under treatment after 2 h. Administration was completed slowly thereafter.

### Haematological toxicity

The DLT was haematological for patients treated in arm A and B without G-CSF support (Table 3). The principal toxicity was leucopenia and neutropenia, although thrombocytopenia was dose-limiting for one patient treated at 190 mg m^−2^ in Arm B+G-CSF. The median time (range) to recovery of neutrophils (>1 × 10^9^ l^−1^) for each arm over the whole range of administered doses were: Arm A 23 days (14–28), Arm B 25 days (19–26), Arm B+G-CSF 18 days (0–24).

The MTD of DaunoXome was 155 mg m^−2^ in Arm A and 100 mg m^−2^ in arm B. Recruitment of patients in Arm B with G-CSF support was suspended at a time when two patients had been enrolled at the 190 mg m^−2^ level, one of whom had shown grade IV haematological toxicity.

Haematological toxicity was not generally greater following second or subsequent doses of DaunoXome. Greater CTC toxicity was seen in only in only two patients.

### Cardiac toxicity

**Figure 1:**
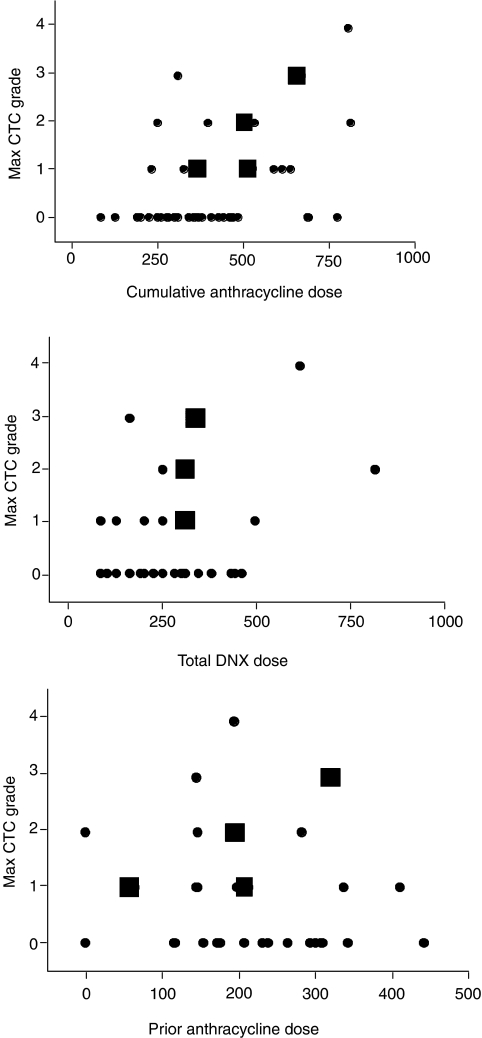
Cardiac toxicity by anthracycline exposure. Data displayed in red refers to patients who had received prior radiotherapy to the chest. Doses are in mg m^−2^. DNX=DaunoXome.

An unexpectedly high rate of cardiac toxicity – 14/48 patients – was found. An additional patient experienced cardiac toxicity following a second ‘off study’ course of DaunoXome. Of these 15 patients, seven experienced CTC grade I, five grade II, two grade III and one grade IV. There was no cardiac toxicity seen in patients who received a cumulative dose (native anthracyclines and DaunoXome) of <240 mg m^−2^ anthracycline, but no correlation beyond this was seen between the grade of toxicity and total exposure to anthracycline in this study (Figure 1). A summary of cardiac toxicity is given in Table 4.

In 13/15 cases, grade 1–IV cardiac toxicity was thought to be at least possibly related to the study drug. In one patient, toxicity was probably due to a symptomatic pericardial effusion, attributable to underlying disease. One other patient had a pericardial effusion (grade I toxicity) unrelated to DaunoXome.

In all, 13 of 15 patients had received prior anthracyclines, with cumulative doses from 60 to 420 mg m^−2^ (median 200 mg m^−2^). In all, 15 of 30 patients evaluable for cardiac toxicity who did NOT develop cardiac toxicity, had received previous anthracycline (dose range 117–450, median 243 mg m^−2^). There was a trend towards increased toxicity in the group with prior anthracycline exposure (*P*=0.0523, Fisher's Exact test).

In all, 10 of 15 patients with cardiac toxicity had previously received radiotherapy (three directly to the chest and one had total body irradiation). Four patients without cardiotoxicity had received prior radiotherapy to the chest, and one had received TBI. There was no statistically significant difference in cardiac toxicity between these two populations (*P*=0.40, *χ*^2^ test). The small numbers for patients treated with prior radiotherapy to the chest meant that no statistical difference could be identified (OR=2.4, 95% CI: 0.5–11.2).

Three patients experienced unrelated or transient cardiac abnormalities at the end of cycle 1. One patient showed a fall in Fractional Shortening (FS) from 38 to 29% after a single dose of 80 mg m^−2^. This patient was reported to have normal cardiac function, but had a pericardial effusion secondary to progressive metastatic disease. This change was considered to be related exclusively to disease. One patient showed a fall of FS from 35 to 28% (20% reduction) after a single dose of 80 mg m^−2^. The echocardiogram was reported to be otherwise normal, and one week later, repeat echocardiography showed FS to be 36%. This change in function was felt to be a drug-related toxicity but was transient. One further patient suffered a cardiac DLT after a single dose of 125 mg m^−2^, with a reduction in Ejection Fraction (EF) by more than 20% (74 to 54%). A repeat echocardiogram one week later showed normal cardiac function, (FS 40%, EF 72%), and the treating physician elected to continue with DaunoXome off-study on a compassionate basis. Cardiac function after this cycle remained normal. A further cohort of patients was recruited at the same dose level, with no further cardiac toxicity.

The results from these three patients prompted a review of criteria for the assessment of cardiac toxicity. There was a concern that transient falls in measured cardiac function were not representative of true changes in function, and that an inappropriately low MTD might therefore be identified. A protocol modification was made, such that cardiac toxicity would be evaluated at 21 days and, if abnormal, a second evaluation would be made at day 28. Only if cardiac function were still abnormal, measured by an absolute FS of <29% or a change in EF by ⩾20%, would cardiac DLT be reportable.

In fact, no further patient developed a cardiac DLT after the first cycle of DaunoXome, at day 21 or day 28, and this modification to the protocol did not, therefore, affect the outcome of the study. Significant cardiac toxicity was identified, however, in three patients who received repeated courses of DaunoXome. One patient, who had received no prior anthracycline chemotherapy, developed a reduced EF (a fall of 28% from 80 to 58%) after eight cycles, and a cumulative anthracycline dose of 825 mg m^−2^. The ejection fraction recovered to 63% 1 week later, and the patient showed full recovery 1 month later (EF 70%). No further anthracycline was given. This patient eventually died of disease progression 3 years after entry into the study.

One patient, who had received radiotherapy to the left lung and spine, and a prior anthracycline dose of 328 mg m^−2^, received four cycles of DaunoXome without cardiac toxicity. After the fourth cycle, a rapid, and eventually fatal deterioration in cardiac function developed. Autopsy confirmed cardiomyopathy consistent with anthracycline toxicity. This patient received a cumulative anthracycline dose of 668 mg m^−2^ (340 mg m^−2^ of which was DaunoXome).

One patient developed rapidly progressive cardiac impairment after four cycles of DaunoXome (dose level 155 mg m^−2^), and died as a result of cardiac failure. This patient had received a cumulative anthracycline dose of 818 mg m^−2^ (620 mg m^−2^ of which was DaunoXome).

As a result of these two deaths in patients with repeated dosing, recruitment into the study was suspended, pending review of patient data. The trial was subsequently closed before the conventionally defined MTD had been identified for patients with G-CSF support in Arm B.

### Pharmacokinetics

**Figure 2:**
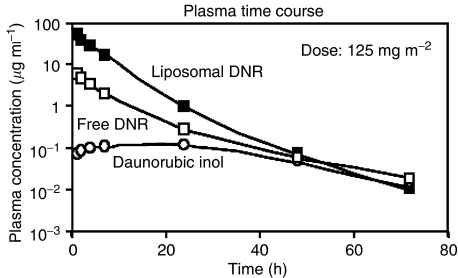
Typical pharmacokinetic profiles for each anthracycline species.

Pharmacokinetic analysis was performed for a total of 31 patients, (five at 80 mg m^−2^, five at 100 mg m^−2^, eight at 125 mg m^−2^, nine at 155 mg m^−2^, four at 190 mg m^−2^). The AUCs of the three compounds were evaluated by the trapezoidal rule. Pharmacokinetic parameters are given in Table 5. Typical pharmacokinetic profiles for each anthracycline species are given in Figure 2.

Measured parameters for DaunoXome were similar to those reported previously (Bellott *et al*, 2001). Liposomal Daunorubicin showed one-compartment elimination kinetics in all patients, and parameters were not dose-dependent. Total plasma clearance was (mean±s.d.): 0.423±0.220 l h^−1^ m^2^, the elimination half-life 5.55±1.07 h, and the total volume of distribution 3.47±2.49 l m^−2^.

Free daunorubicin (DNR) was seen in plasma in most patients at the end of DaunoXome infusion, although the peak concentration (*C*_max_) was delayed by 2–4 h in 10 patients. It had essentially the same pharmacokinetic behaviour as free administered daunorubicin. Free daunorubicin followed biexponential elimination kinetics in most patients; in six, a mono-exponential curve better described the observed data. The elimination half-life of free daunorubicin was independent from the dose administered. Its mean value in the overall population was 11.3±3.9 h.

Daunorubicin was converted to daunorubicinol which reached its maximal plasma concentration 7–24 h after the onset of DaunoXome infusion and was eliminated with mono-exponential decay with a half-life of 17.6±5.2 h, independent of administered dose.

No significant difference in the pharmacokinetic parameters of the three compounds was exhibited between patients included in arm A or in arm B.

### Relationships between AUC and dose

**Figure 3:**
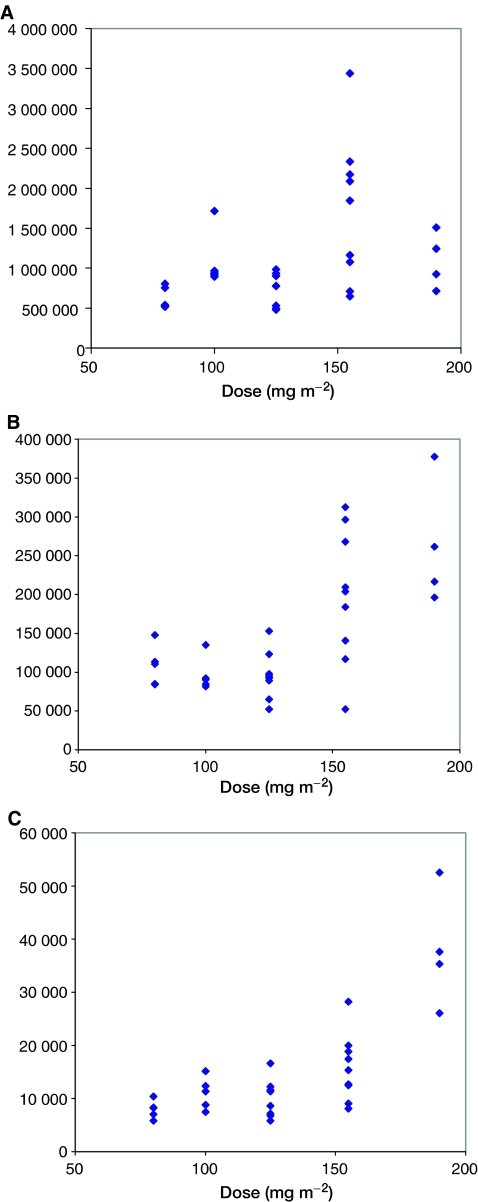
Total exposure to Liposomal and free Daunorubicin, and to Daunorubicinol as a function of administered dose of DaunoXome (mg m^−2^).

**Figure 4:**
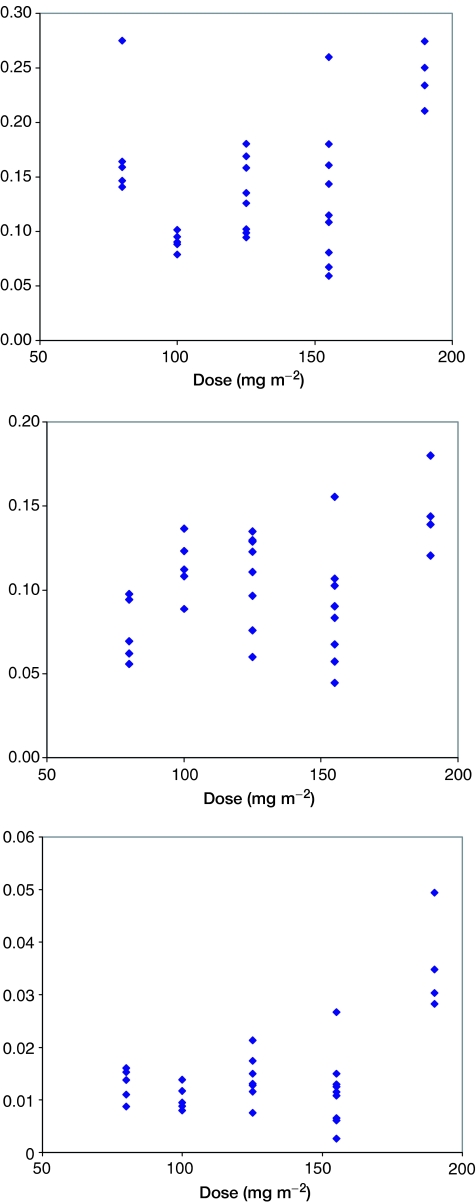
AUC ratios for (**A**) Daunorubicin to Daunoxome, (**B**) Daunorubicinol to Daunorubicin and Daunorubicinol to DaunoXome, as a function of administered DaunoXome dose.

The AUCs of the three compounds analysed are shown as a function of the dose administered in Figure 3. DaunoXome AUC correlated only poorly with administered dose, and no significant difference in AUCs of liposomal daunorubicin, free daunorubicin and daunorubicinol was seen at doses of 80, 100 and 125 mg m^−2^ (Figure 4).

The ratio of AUCs of free daunorubicin to liposomal daunorubicin varied between 0.1 and 0.2 in most patients. A statistically greater conversion of liposomal to free daunorubicin was seen at the highest dose (190 mg m^−2^). This higher AUC of free drug seen at higher doses might be expected to give a nonlinear risk of cardiac toxicity. The AUC ratio of daunorubicinol to free daunorubicin varied for most patients between 0.05 and 0.12, and once again, greater conversion of daunorubicin to daunorubicinol was seen at the highest dose.

There was no relationship between the pharmacokinetic parameters evaluated in these patients and their age; in addition, the pharmacokinetic parameters in those patients who developed cardiac toxicity or who responded to DaunoXome therapy were close to median values for the population as a whole and could not explain these clinical events.

### Patient response to treatment

Tumour response was assessed after two cycles in 29 patients. Response was measured by MRI in all cases. One patient was not evaluable, six had nonmeasurable disease, seven had SD, 14 had PD. One relapsed Primitive Neuro-Ectodermal tumour had a partial response, which continued for four cycles. One patient with ependymoma had stable disease for eight cycles of DaunoXome, but developed cardiac toxicity (grade II). He died from progressive disease, 3 years after entry into the study (Table 6).

### Patient withdrawal from study

In all, 39 patients were withdrawn from study due to treatment failure, three because of cardiac adverse events, one because of neurological deterioration (PD), one because they died (PD). One patient was withdrawn so that drug could be given compassionately at a different hospital, one to have surgery, one because of a protocol violation and one because of drug shortage.

## DISCUSSION

The development of new therapies in paediatric oncology remains slow for several reasons. There is a limited supply of new drugs suitable for development. Due to small numbers of patients, phase I and II studies are slow to run, and ethical concerns may lead to reluctance to enroll young patients. It is clear, however, that in childhood, toxicities may be different from those in adults (Doz *et al*, 2001) and that the tolerance of children may be better(Vassal *et al*, 2003). When a DLT has been defined in adults, it should be also defined in a paediatric cohort.

Paediatric tumours differ histologically from adult tumours, and appear to have greater sensitivity to chemotherapy. Timing of phase I studies in children should therefore be early, not dependent upon results from adult studies. It is to be hoped that new regulations in the USA and Europe will encourage early release of new agents by industry.

Anthracyclines have demonstrated efficacy in most paediatric tumours. Cardiotoxicity is a major concern in children, given the expected growth of the child, and long post-treatment survival anticipated. The reduction of cardiac toxicity of these very active compounds would be of great benefit.

A criticism of this study might be that patients had received prior anthracyclines, and that the cardiac toxicity identified does not represent the true risk associated with DaunoXome. While this is a valid criticism, it would be unusual for patients who reach this stage of treatment to be anthracycline-naïve, particularly in the group who have already undergone high-dose therapy. Recruitment would of necessity show a predominance of brain tumours, as in the study of Lippens *et al* (Lippens, 1999). Given the encouraging reports suggesting lack of cardiac toxicity with this agent, it was therefore felt appropriate to include all potentially eligible patients.

The assessment of cardiotoxicity is not well defined: there is only poor correlation between acute and delayed cardiotoxicity, and the significance of acute changes is therefore unknown. Cardiac troponins have been proposed as markers of acute myocardial damage, but their value in prediction of outcome is still uncertain (Missov *et al*, 1997; Sparano *et al*, 2002; Auner *et al*, 2003). The assessment of cardiac toxicity within a phase I study is therefore problematic, and there is no guidance in published literature. Within the design of a phase I trial, given that patients usually have end-stage disease, long-term follow-up is rarely considered. It is possible that the reports from adult studies of DaunoXome, of no cardiotoxicity even with high cumulative doses, may therefore be misleading.

In this study, we report that liposomal anthracyclines have an unexpectedly high rate of cardiotoxicity in children. Whether cardiotoxicity would appear in anthracycline-naive patients, and whether higher doses of a liposomal compound as compared to naive compound may be delivered remains a matter of debate.

The dose-limiting toxicity of DaunoXome without G-CSF is neutropenia, at 155 and 100 mg m^−2^ for patients with conventional or heavy previous treatment, respectively. Recruitment to Arm B with G-CSF support was halted having recruited two patients at 190 mg m^−2^, with one DLT (thrombocytopenia). This was not unexpected for the group of patients under study. In all, 30 out of 43 patients experienced grade IV neutropenia during the first cycle of treatment. The most significant fall in neutrophil count occurred between 10 and 19 days following administration of DaunoXome, but recovery was usual by day 21.

The noncardiac, nonhaematological toxicities reported were related to myelosuppression (febrile neutropenia), but nausea, hypermagnesaemia and allergic reaction to DaunoXome were also seen. These toxicities were not considered excessive for patients undergoing this treatment regime.

This is the first paediatric study with liposomal Daunorubicin that has shown a significant degree of cardiac toxicity, albeit with repeated dosing. Three other paediatric studies have been performed, but none of these raised concerns over the degree of cardiac toxicity (Baruchel, personal communication; Lippens, 1999; Hempel *et al*, 2003). The treatment regimes used in these studies differ significantly from the present study: in both, patients were given four doses over a 4-week time period. Patients treated in the study of Baruchel received individual doses up to 120 mg m^−2^, to a maximum cumulative dose of 720 mg m^−2^. In the study of Lippens (1999), patients received individual doses of 60 mg m^−2^, to a maximum cumulative dose of 600 mg m^−2^. In the study reported by Hempel *et al*, patients received individual doses of 30–60 mg m^−2^ on days 1 and 5 of each cycle. In the present study, patients received a single dose every 21 days, up to a maximum of eight cycles. The two patients who died as a result of DaunoXome treatment both had high cumulative levels of anthracyclines (668 and 818 mg m^−2^), but not markedly different from those previously reported.

### Comparison of PK

A response rate of about 4% is often seen in phase I studies, and where responses occur more frequently, activity of the agent is felt to be worth exploring further. In this study, only one patient demonstrated a partial response, after two and four cycles. One other demonstrated a prolonged period of stable disease, for eight cycles of chemotherapy. The response rate of one of 48 patients (2.1%) was disappointing.

## CONCLUSION

Although it was hoped that cardiac toxicity with DaunoXome would be significantly reduced compared to conventional Daunorubicin, this was not seen. Studies in anthracycline naïve patients may still be justified, but severe cardiotoxicity in patients receiving repeated courses of DaunoXome mean caution should be exercised in the use of this drug in children. The possibility that DaunoXome pharmacokinetics may change, such that a greater exposure to anthracycline or its active metabolite is seen at higher doses, may account for the observed cardiac toxicity in this study, but further confirmation of this is required.

## Figures and Tables

**Table 1:** DaunoXome patient characteristics

	**Arm A (no G-CSF)**	**Arm B (no G-CSF)**	**Arm B (with G-CSF)**	**Total numbers**
Total number entered	27	13	8	48
Total number evaluable for cardiac toxicity	25	12	8	45
Total number evaluable for haematological toxicity	24	12	7	43
No. of males	14	8	6	28
No. of females	13	5	2	20
				
Age (years) median (Range)	10.0 (1.3–18.4)	8.5 (1.9–18.5)	9.7 (4.2–11.1)	9.6 (1.3–18.5)
				
Diagnosis	Osteosarcoma 5 Rhabdomyosarcoma 2 PNET 2 Glioma 5 Ewings 1 Astrocytoma 2 Ependymoma 2 NHL 1 Neurosarcoma 1 Hodgkins 1 Undiff nasopharngeal ca 1 Hepatocellular carcinoma 1 Malignant germinal tumour 1 Malignant adrenal cortical carcinoma 1 Choroid plexus carcinoma 1	Neuroblastoma 4 Synovial sarcoma 2 Medulloblastoma 1 Spinal PNET 1 Osteosarcoma 1 Ewings 1 PPNET 1 Hodgkins 1 Anaplastic Ependymoma 1	Neuroblastoma 4 Clear cell sarcoma 1 Rhabdomyosarcoma 1 Medulloblastoma 1 Nephroblastoma 1	
				
*Prior therapy*				
Chemotherapy^a^	22	12	8	42
Radiotherapy	17	6	4	27
Surgery	21	11	8	40
No. of patients previously treated with anthracyclines	15	8	8	31
Median prior anthracyclines dose (mg)	0	88	224	
No. of patients who previously had RT that affected the chest	4	2 (1 TBI)	3 (1 TBI)	9

a May have had more than one previous chemotherapy treatment. Abbreviation: TBI, Total body irradiation.

**Table 2:** Non-haematological toxicities

**Toxicity**	**Episodes**	**Patients**
Fever or infection	30	24
Nausea and/or vomiting	19	16
Rash/bruising	5	3
Alopecia	2	2
Allergic reaction to DaunoXome	2	2
Mucositis	3	3
Biochemical change	31	11
Pain	9	7
Other	24	16
Total		48

Data for all cycles (*n*=98) are included.

**Table 3:** Haematological toxicity – cycle 1 data only

		**Maximum toxicity grade reported**							
		**Neutrophils**		**Leukocytes**		**Platelets**		**Haemoglobin**	
**Dose (mg m^−2^)**	**No. evaluable/No. courses**	**3**	**4**	**3**	**4**	**3**	**4**	**3**	**4**
*Arm A*									
80	2/2	1	0	1	0	0	1	1	0
100	3/3	1	1	0	1	0	0	0	0
125	7/8	3	4	5	2	1	1	2	0
155	6/7	0	6	3	3	1	0	0	0
190	6/7	0	6	0	6	2	1	3	1
									
*Arm B*									
80	3/3	1	2	2	0	2	0	1	0
100	7/7	2	5	2	3	2	3	4	1
125	2/3	0	2	0	2	0	1	0	0
									
*Arm B*+*G-CSF*									
125	3/3	0	1	0	1	1	2	0	0
155	3/3	1	2	3	0	0	2	1	0
190	1/2	0	1	0	1	0	1	0	0

Numbers refer to the maximum grade of toxicity experienced, but duration is not considered.

**Table 4:** Summary of cardiac toxicity identified

		**Baseline**							
**Patient number**	**Dose (mg m^−2^)**	**FS**	**EF**	**Course number**	**CTC grade**	**Toxicity**	**Causality**	**Resolution?**	**Previous anthracyclines/Chest RT**
1	80	35		1	I	Day 21 FS=28%	Possibly	Day 28, FS=36%, overall function ‘good’	Doxorubicin 150 mg m^−2^
3	80	38	76	1	I–II	Day 21 small pericardial effusion	Possibly	Proceeded to second course	Doxorubicin 148.6 mg m^−2^
				2	III	Cycle 2 day 24 Echo FS=26%, EF=59%	Unlikely	No. Effusion related to metastatic disease.	
7	125	42	74	1	II	Day 21 FS=27%, EF=54% (drop of 27%)	Related	Yes. Day 28 FS=40% EF=72%	Doxorubicin 150 mg m^−2^
13	125	40	70	1	I	Day 21 EF=57% (fall of 18.6%)	Related	Yes. Day 28, FS=36%, EF=74%	Doxorubicin 344 mg m^−2^
14	125	39	77	1	II	Day 21 EF=61% (fall of 20.1%	Related	Yes. Day 36 FS=40%, EF=70%	No
19	155	30	66	2	II	Day 21 cycle 2 23.10.00 FS=30% EF=67% 8.11.00 FS=27.5%, EF=62% 15.11.00 FS=20.18%, EF=49%	Related	No further echo	Doxorubicin 200 mg m^−2^ 20 Gy RT to chest
21^a^	155			4	IV	Cycle 4 EF 24%, FS 24%	Related	No	Epiadriamycin 198 mg m^−2^
25	190	32	66	3	I	Day 21 FS=29%	Probable	Yes. Day 28 FS=30%	Doxorubicin 147 mg m^−2^
104	100, 80 (X3)	30	63	4	IV	Cycle 4 FS=20%, EF=49%	Probable	No. Patient died: congestive cardiac failure	Adriamycin 328 mg m^−2^, 45 Gy to spine and left lung
107	100	30	67	1	I	Small pericardial effusion	Unlikely	Yes. Echo subsequently normal FS 41% EF 73%	Doxorubicin 420 mg m^−2^
114	125, 100 (X6)	49	80	8	II	Cycle 8, day 21 FS=25%, EF=58%	Related	No	No
112	125	32	60	1	I	Cycle 1, day 21 FS=27%, EF=53%	Probable	Patient withdrawn from study	Doxorubicin 202 mg m^−2^
302	125+GCSF	37	68	2^a^	II	Cycle 2, day 21 FS=25%, EF=50%	Possibly	Progressive disease, no further echo. No clinical symptoms	Adriamycin 288 mg m^−2^
304	155+GCSF	34	63	2	I	Cycle 2, day 21 FS=29%	Probable	Yes	Doxorubicin 61 mg m^−2^, Epirubicin 150 mg m^−2^
305	155+GCSF	34	63	1	I	Cycle 1, day 21 FS=29%	Probable	No further echo	Idarubicin 10 mg m^−2^, Total body irradiation (12 Gy)

a Off study.

*Abbreviations*: FS, Fractional shortening. EF, Ejection Fraction.

**Table 5:** Pharmacokinetic parameters

	**80**		**100**		**125**		**155**		**190**		**Overall**	
**Dose (mg m^−2^)**	**Mean**	**s.d.**	**Mean**	**s.d.**	**Mean**	**s.d.**	**Mean**	**s.d.**	**Mean**	**s.d.**	**Mean**	**s.d.**
*AUC ((ng ml*^−*1*^) × *h*)												
DaunoXome	628 521	139 129	108 7906	352 553	734 347	206 424	171 9306	907 368	109 7766	349 420		
Daunorubicin	108 206	26 103	96 802	21 902	95 966	31 363	198 159	86 022	262 881	81 111		
Daunorubicinol	7967	1700	11043	3021	10033	3592	15 803	6219	37 885	10 944		
												
*Clearance (l h m*^−*2*^)												
DaunoXome	0.46	0.35	0.26	0.06	0.50	0.12	0.36	0.19	0.57	0.27	0.42	0.22
												
*Elimination half-life (h)*												
DaunoXome	6.94	1.49	5.36	0.23	4.93	0.47	5.25	1.05	5.93	0.46	5.55	1.07
Daunorubicin	12.63	3.82	10.22	1.50	10.94	2.81	10.13	4.17	14.66	6.35	11.34	3.90
Daunorubicinol	20.03	7.51	15.95	2.31	17.47	3.86	19.04	7.05	14.29	2.51	17.59	5.24
												
*Volume of distribution (l m*^−*2*^)												
DaunoXome	4.99	5.04	2.00	0.41	3.53	1.01	2.71	1.55	4.99	2.59	3.47	2.49
												
*AUC ratio*												
Daunorubicin: DaunoXome	0.18	0.06	0.09	0.01	0.13	0.03	0.13	0.06	0.24	0.03	0.15	0.06
Daunorubicinol: Daunorubicin	0.08	0.02	0.11	0.02	0.11	0.03	0.09	0.03	0.15	0.02	0.10	0.03

**Table 6:** Previously reported pharmacokinetics

				**T1/2 (h)**	**Clp (l h^−1^ per m^2^)**	**Vd (l m^−2^)**	**AUC ratio (Dol:DNR)**
**Author**	**Year**	**Patient numbers**	**Dose (mg m^−2^)**	**Mean±s.d.**	**Mean±s.d.**	**Mean±s.d.**	**Mean**
Guaglione	1994	32	10–60	4.67	0.363	1.79	
Gill	1995	40	10–80	4.82	0.337	1.81	
Fumigalli	2000						
DNX alone		6	40	5.6±2.6	0.39±0.17	3.2±1.6	
DNX+Indinavir		9	40	5.8±2.1	0.5±0.36	4.2±1.4	
DNX+ritonavir		6	40	6.5±3.9	0.46±0.2	4.3±1	
Pea	2000	11	60	4.54±0.9	0.47±0.26	2.88±0.9	0.041±0.015
Bellot	2001	18	40–120	5.23±1	0.34±0.22	2.08±0.7	0.815±0.412
Hempel	2003	24	60	5.66	0.233	1.93	
Present study		31	80–190	5.55±1.07	0.422±0.22	3.47±2.49	0.10±0.03
							
					l h^−1^		
Yeo	1999	21	100	1.81±1.3 (*α*)	0.90±0.33		0.072±0.039
				7.36±2.7 (*β*)			
